# Supplementary material for: Selection for High Oridonin Yield in the Chinese Medicinal Plant Isodon (Lamiaceae) Using a Combined Phylogenetics and Population Genetics Approach
Source: PLoS One. 2012 Nov 27;7(11):e50753. doi: 10.1371/journal.pone.0050753 (PMC3507737; doi:10.1371/journal.pone.0050753)
Supplement: Table S2 — Summary of literature reports of oridonin as detected in species of Isodon . (DOC) [file pone.0050753.s002.doc]

| **Species** | **Oridonin Production (A = Absent; P= Present; A/P = both reported)** | **References** |
| --- | --- | --- |
| *adenanthus* | A | [1–5] |
| *adenolomus* | A | [3,6,7] |
| *amethystoides* | A/P | [3,8] |
| *bulleyanus* | A | [3] |
| *calcicola* | A | [3,9] |
| *coetsa* | A | [3,10–12] |
| *effusus* | A | [3] |
| *enanderianus* | A | [3,13–18] |
| *eriocalyx* | A/P | [3,19–26] |
| *excisus* | A | [3,27–31] |
| *flavidus* | A | [3,32] |
| *flexicaulis* | A | [3,33] |
| *forrestii* | A | [3,34–36] |
| *gesneroides* | A | [3,37,38] |
| *glutinosus* | A | [3,39] |
| *grandifolius var. atuntzeensis* | A | [3] |
| *henryi* | A | [3,40,41] |
| *inflexus* | A | [3] |
| *irroratus* | A | [3] |
| *japonicus* | A/P | [3,42–46] |
| *japonicus var. glaucocalyx* | A/P | [3,47,48] |
| *leucophyllus* | A/P | [3,49–52] |
| *longitubus* | P | [3] |
| *lophanthoides* | A | [3] |
| *lophanthoides var. gerardianus* | A | [3,53,54] |
| *loxothyrsus* | A | [3,7,55,56] |
| *lungshengensis* | A | [3] |
| *megathyrsus* | A | [3,57] |
| *melissoides* | A | [3,58–61] |
| *nervosus* | A/P | [3,62–65] |
| *pharicus* | A | [3,66–68] |
| *phyllostachys* | A/P | [3,69–73] |
| *pleiophyllus* | A | [3,7] |
| *rosthornii* | A/P | [3,74,75] |
| *rubescens* | P | [3,76–81] |
| *rugosus* | A | [3] |
| *scoparius* | A | [82,83] |
| *sculponeatus* | A | [3,84–87] |
| *shikokianus var. intermedius* | A | [3] |
| *shikokianus var. shikokianus* | P | [3] |
| *shikokianus var. occidentalis* | A | [3] |
| *ternifolius* | A/P | [3,88–90] |
| *trichocarpus* | P | [3,42,43] |
| *umbrosus var. umbrosus* | A | [3] |
| *umbrosus var. latifolius* | A | [3] |
| *umbrosus var. leucanthus* | A | [3] |
| *umbrosus var. hakusanensis* | A | [3] |
| *wikstroemioides* | A | [3,91] |
| *xerophilus* | A | [3,92–96] |
| *yuennanensis* | A | [3] |

**References**

1. Jiang B, Xu JC, Yang H, Zhao QS, Sun HD (2000) Two new ent-kaurane diterpenoids from *Isodon adenantha*. Chin Chem Lett 11: 995–996.

2. Jiang B, Yang H, Li ML, Hou AJ, Han QB, et al. (2002) Diterpenoids from *Isodon adenantha.* J Nat Prod 65: 1111–1116.

3. Sun HD (2001) Diterpenoids from Isodon species (香茶菜属植物二萜化合物). Beijing: Science Publishing House. 387 p.

4. Xiang W, Han QB, Li SH, Na Z, Sun HD (2003) Ent-kaurene diterpenoids from *Isodon adenanthus*. Acta Bot Sin 45: 1383–1386.

5. Xiang W, Song QS, Zhang HJ, Li RT, Na Z, et al. (2004) Adenanthusone, a new ursane type nortriterpenoid from *Isodon adenanthus*. Helv Chim Acta 87: 2842–2847.

6. Ping ZR, Zhang HJ, Lin ZW, Zhen YL, Sun HD (1992) Diterpenoids from *Isodon adenoloma*. Phytochemistry 31: 4237–4240.

7. Sun HD, Lin ZW, Niu FD, Lin LZ, Chai HB, et al. (1995) Cytotoxic ent-kaurene diterpenoids from 3 *Isodon* species. Phytochemistry 38: 437–442.

8. Jin YR, Du YF, Shi XW, Liu PW (2010) Simultaneous quantification of 19 diterpenoids in *Isodon amethystoides* by high-performance liquid chromatography-electrospray ionization tandem mass spectrometry. J Pharm Biomed Anal 53: 403–411.

9. Chen SN, Cehn S, Lan H, Lin ZW, Sun HD, et al. (1998) Diterpenoids from *Isodon calcicola* var*. subculva*. Phytochemistry 49: 2437–2441.

10. Xu YL, Kubo I (1993) Diterpenoid constituents from *Rabdosia coetsa*. Phytochemistry 34: 576–578.

11. Li WW, Li BG, Ding LS, Chen YZ (1998) The chemical constituents of *Rabdosia coetsa*. Acta Bot Sin 40: 461–465.

12. Liu YH, Chia LS, Ding JK, Shen YH, Li RT, et al. (2006) Two new rearranged abietane diterpenoids from tropical *Isodon coetsa*. J Asian Nat Prod Res 8: 671–675.

13. Wang YH, Chen YZ, Lin ZW, Sun HD (1998) A new 1 beta-hydroxy-ent-kaurenoid from *Isodon enanderianus*. Chin Chem Lett 9: 733–734.

14. Wang YH, Chen YZ, Lin ZW, Sun HD, Fan JS (1998) 7,20-epoxy-ent-kaurenoids from *Isodon enanderianus*. Phytochemistry 48: 1267–1269.

15. Na Z, Jiang B, Yang H, Lin ZW, Sun HD (2001) A new ent-kaurane diterpenoid from *Isodon enanderianus*. Chin Chem Lett 12: 711–712.

16. Na Z, Xiang W, Niu XM, Mei SX, Lin ZW, et al. (2002) Diterpenoids from *Isodon enanderianus*. Phytochemistry 60: 55–60.

17. Na Z, Li SH, Xiang W, Zhao AH, Li CM, et al. (2002) A novel asymmetric ent-kauranoid dimer from *Isodon enanderianus*. Chin J Chem 20: 884–886.

18. Xiang W, Na Z, Li SH, Li ML, Li RT, et al. (2003) Cytotoxic diterpenoids from *Isodon enanderianus*. Planta Med 69: 1031–1035.

19. Shen XY, Isogai A, Furihata K, Kaniwa H, Sun HD, et al. (1989) Ent-kaurene diterpenoids from *Rabdosia eriocalyx*. Phytochemistry 28: 855–858.

20. Sun HD, Lin ZW, Niu FD, Shen PQ, Pan LT, et al. (1995) Diterpenoids from *Isodon eriocalyx* var. *laxiflora*. Phytochemistry 38: 1451–1455.

21. Wang J, Lin ZW, Zhao QS, Sun HD (1998) Diterpenoids from *Isodon eriocalyx*. Phytochemistry 47: 307–309.

22. Chen NY, Tian MQ, Wu R, Sun HD, Li CM, et al. (2000) Diterpenoids from *Isodon eriocalyx*. J Chin Chem Soc 47: 363–366.

23. Niu XM, Li SH, Li ML, Zhao QS, Mei SX, et al. (2002) Cytotoxic ent-kaurane diterpenoids from *Isodon eriocalyx* var. *laxiflora*. Planta Med 68: 528–533.

24. Niu XM, Li SH, Zhao QS, Mei SX, Lin ZW, et al. (2003) Novel ent-abietane diterpenoids from *Isodon eriocalyx* var. *laxiflora*. Helv Chim Acta 86: 299–306.

25. Shen YH, Wen ZY, Xu G, Xiao WL, Peng LY, et al. (2005) Cytotoxic ent-kaurane diterpenoids from *Isodon eriocalyx*. Chem Biodivers 2: 1665–1672.

26. Li XN, Pu JX, Du X, Lou LG, Li LM, et al. (2010) Structure and cytotoxicity of diterpenoids from *Isodon eriocalyx*. J Nat Prod 73: 1803–1809.

27. Chang RG, Kim DS, Zeduk U, Shen XY, Chen YP, et al. (1992) A diterpenoid from *Rabdosia excisa*. Phytochemistry 31: 342–343.

28. Kim HK, Whang WK, Kim IH (1997) Constituents of the herb of *Isodon excisus* var. *coreanus*. Arch Pharm Res 20: 291–296.

29. Gui MY, Aoyagi Y, Jin YR, Li XW, Hasuda T, et al. (2004) Excisanin H, a novel cytotoxic 14,20-epoxy-ent-kaurene diterpenoid, and three new ent-kaurene diterpenoids from *Rabdosia excisa*. J Nat Prod 67: 373–376.

30. Hong SS, Lee SA, Han XH, Jin HZ, Lee JH, et al. (2007) Kaurane diterpenoids from *Isodon excisus* inhibit LPS-Induced NF-kappa B activation and NO production in macrophage RAW264.7 cells. J Nat Prod 70: 632–636.

31. Hong SS, Lee SA, Kim NY, Hwang JS, Han XH, et al. (2011) Pyrrolidinone diterpenoid from *Isodon excisus* and inhibition of nitric oxide production in lipopolysaccharide-induced macrophage RAW264.7 cells. Bioorg Med Chem Lett 21: 1279–1281.

32. Zhao QS, Tian J, Yue JM, Chen SN, Lin ZW, et al. (1998) Diterpenoids from *Isodon flavidus*. Phytochemistry 48: 1025–1029.

33. Zhang HJ, Sun HD (1989) Diterpenoids from *Rabdosia flexicaulis*. Phytochemistry 28: 3534–3536.

34. Xu YL, Kubo I, Tang CS, Zhang FL, Sun HD (1993) Diterpenoids from *Rabdosia forrestii*. Phytochemistry 34: 461–465.

35. Mei SX, Deng YX, Han QB, Lin ZW, Sun HD (2001) A new diterpene glycoside from *Isodon forrestii*. Chin Chem Lett 12: 145–146.

36. Kubo I, Shimizu K, Xu YL (2003) Ent-isopimarane diterpenoids of the leaves from *Rabdosia forrestii*. Fitoterapia 74: 643–649.

37. Sun HD, Lin ZW, Niu FD, Zhen QT, Wu B, et al. (1995) Cytotoxic ent-kaurene diterpenoids from *Isodon gesneroides*. Phytochemistry 40: 1461–1467.

38. Chen SN, Zhang HJ, Lin ZW, Chen YZ, Sun HD (1998) Diterpenoids from *Isodon gesneroides.* Phytochemistry 48: 155–158.

39. Huang H, Chen YP, Zhang HJ, Lin ZW, Zhao SX, et al. (1997) Diterpenoids from *Isodon glutinosus*. Phytochemistry 45: 559–562.

40. Meng XJ, Chen YZ, Cui YX, Cheng JL (1988) Diterpenoids from *Rabdosia henryi*. J Nat Prod 51: 812–816.

41. Zhao Y, Huang SX, Yang LB, Pu JX, Xiao WL, et al. (2009) Cytotoxic ent-kaurane diterpenoids from *Isodon henryi*. Planta Med 75: 65–69.

42. Fujita E, Fujita T, Shibuya M (1966) Diterpenoid constituents of *Isodon trichocarpus* and *Isodon japonicus* (Terpenoids 4). Tetrahedron Lett 27: 3153.

43. Fujita E, Fujita T, Katayama H, Shibuya M, Shingu T (1970) Terpenoids. 15. Structure and absolute configuration of oridonin isolated from *Isodon japonicus* and *Isodon trichocarpus*. J Chem Soc C 12: 1674.

44. Fujita E, Fujita T, Katayama H, Shibuya M (1967) Oridonin a new diterpenoid from *Isodon* Species. Chem Commun 6: 252.

45. Yang LB, Huang SX, Li LM, Zhao Y, Lei C, et al. (2007) Ent-kaurane diterpenoids from *Isodon japonicus*. Helv Chim Acta 90: 2375–2379.

46. Hong SS, Lee SA, Han XH, Hwang JS, Lee C, et al. (2008) Ent-kaurane diterpenoids from *Isodon japonicus*. J Nat Prod 71: 1055–1058.

47. Meng XJ, Wang QG, Chen YZ (1989) Diterpenoids from *Rabdosia henryi*. Phytochemistry 28: 1163–1165.

48. Kim DS, Chang RG, Shen XY, Chen YP, Sun HD (1992) Diterpenoids from *Rabdosia japonica*. Phytochemistry 31: 697–699.

49. Liao X, Ding LS, Peng SL (1998) Ent-kaurene diterpenoids from *Rabdosia leucophylla*. Phytochemistry 47: 247–250.

50. Chen SN, Lin ZW, Qin GW, Sun HD, Chen YZ (1999) Diterpenoids from *Isodon leucophyllus*. Planta Med 65: 472–474.

51. Zhao AH, Xiang W, Na Z, Wang ZY, Lin ZW, et al. (2004) Cytotoxic ent-kauranoids from *Isodon leucophyllus*. J Asian Nat Prod Res 6: 145–150.

52. Zhang HB, Pu JX, Zhao Y, He F, Zhao W, et al. (2010) Three new 18-oxygenated ent-kaurane diterpenoids from *Isodon leucophyllus*. Nat Prod Commun 5: 1873–1876.

53. Jiang B, Lu ZQ, Zhang HJ, Zhao QS, Sun HD (2000) Diterpenoids from *Isodon lophanthoides*. Fitoterapia 71: 360–364.

54. Li CY, Gao H, Jiao WH, Zhang L, Zhou GX, et al. (2010) Three new diterpenoids from *Rabdosia lophanthoides* var. *gerardiana*. Helv Chim Acta 93: 450–456.

55. Sun HD, Lin ZW, Shen XY, Takeda Y, Fujita T (1991) Ent-kaurene diterpenoids from *Rabdosia loxothyrsa*. Phytochemistry 30: 603–606.

56. Huang H, Sun HD, Zhao SX (1996) Triterpenoids of *Isodon loxothyrsus*. Phytochemistry 42: 1665–1666.

57. Sun HD, Lin ZW, Niu FD, Lin LZ, Chai HY, et al. (1994) Cytotoxic diterpenoids from *Isodon megathyrsus*. J Nat Prod 57: 1424–1429.

58. Zhao QS, Tian J, Yue JM, Chen SN, Lin ZW, et al. (1998) Diterpenoids from *Isodon melissoides*. Phytochemistry 47: 1089–1092.

59. Zhao AH, Han QB, Li SH, Wang FS, Zhao QS, et al. (2003) Four new diterpenoids from *Isodon melissoides*. Chem Pharm Bull 51: 845–847.

60. Zhao AH, Han QB, Li RT, Li SH, Qing C, et al. (2004) Diterpenoids from *Isodon melissoide*s. J Nat Prod 67: 1441–1444.

61. Zhao AH, Li RT, Jiang B, Zhang JX, Zhao QS, et al. (2005) Three new compounds from *Isodon melissoides*. J Asian Nat Prod Res 7: 151–156.

62. Sun HD, Niu FT, Chen YP, Lin ZW (1992) Diterpenoids from *Rabdosia nervosa*. Phytochemistry 31: 695–696.

63. Wang XR, Hu HP, Wang HP, Wang SQ, Ueda S, et al. (1994) Nervosanin-A and nervosanin-B, and ent-kauranoids from *Isodon nervosus*. Phytochemistry 37: 1367–1370.

64. Li LM, Li GY, Ding LS, Yang LB, Zhao Y, et al. (2008) Ent-kaurane diterpenoids from *Isodon nervosus*. J Nat Prod 71: 684–688.

65. Yan FL, Guo LQ, Wang CM, Zhang JX (2009) Chemical constituents of *Isodon nervosus* and their cytotoxicity. J Asian Nat Prod Res 11: 326–331.

66. Wang ZM, Cheng PY, Min ZD, Zheng QT, Wu CY, et al. (1991) Ent-kaurene diterpenoids, isodopharicin-A, isodopharicin-B and isodopharicin-C in *Isodon pharicus*. Phytochemistry 30: 3699–3702.

67. Zhao Y, Huang SX, Xiao WL, Ding LS, Pu JX, et al. (2009) Diterpenoids from *Isodon pharicus*. Tetrahedron Lett 50: 2019–2023.

68. Zhao Y, Pu JX, Huang SX, Ding LS, Wu YL, et al. (2009) Ent-kaurane diterpenoids from *Isodon pharicus*. J Nat Prod 72: 988–993.

69. Hou AJ, Yang H, Jiang B, Zhao QS, Lin ZW, et al. (2000) A new ent-kaurane diterpenoid from *Isodon phyllostachys*. Fitoterapia 71: 417–419.

70. Li X, Xiao WL, Huang SX, Weng ZY, Shen YH, et al. (2006) Structure elucidation of two new diterpenoids from *Isodon phyllostachys*: Phyllostacins A and B. Helv Chim Acta 89: 1181–1186.

71. Li X, Xiao WL, Pu JX, Ban LL, Shen YH, et al. (2006) Cytotoxic ent-kaurene diterpenoids from *Isodon phyllostachys*. Phytochemistry 67: 1336–1340.

72. Li X, Pu JX, Li SH, Huang SX, Weng ZY, et al. (2007) Three new ent-kauranoids from *Isodon phyllostachys*. Heterocycles 71: 2441–2447.

73. Li X, Weng ZY, Li Y, Pu JX, Huang SX, et al. (2008) Ent-kaurane diterpenoids from *Isodon phyllostachys*. Helv Chim Acta 91: 1130–1136.

74. Xu YL, Ma YB (1989) Diterpenoid constituents from *Rabdosia rosthornii*. Phytochemistry 28: 3235–3237.

75. Kubo I, Xu YL, Shimizu K (2004) Antibacterial activity of ent-kaurene diterpenoids from *Rabdosia rosthornii*. Phytother Res 18: 180–183.

76. Li BL, Chen SN, Shi ZX, Chen YZ (2000) Ent-kaurene diterpenoids from *Isodon rubescens*. Phytochemistry 53: 855–859.

77. Han QB, Jiang B, Zhang JX, Niu XM, Sun HD (2003) Two novel ent-kaurene diterpenoids from *Isodon rubescens*. Helv Chim Acta 86: 773–777.

78. Han QB, Li ML, Li SH, Mou YK, Lin ZW, et al. (2003) Ent-kaurane diterpenoids from *Isodon rubescens* var*. lushanensis*. Chem Pharm Bull 51: 790–793.

79. Han QB, Zhao QS, Li SH, Peng LY, Sun HD (2003) Ent-kaurane diterpenoids from *Isodon rubescens* collected in Guizhou Province. Huaxue Xuebao 61: 1077–1082.

80. Han QB, Xiao WL, Shen YH, Sun HD (2004) Ent-kaurane diterpenoids from *Isodon rubescens* var. *rubescens*. Chem Pharm Bull 52: 767–769.

81. Huang SX, Pu JX, Xiao WL, Li LM, Weng ZY, et al. (2007) Ent-Abietane diterpenoids from *Isodon rubescens* var*. rubescens*. Phytochemistry 68: 616–622.

82. Xiang W, Li RT, Song QS, Na Z, Sun HD (2004) Ent-clerodanoids from *Isodon scoparius*. Helv Chim Acta 87: 2860–2865.

83. Zhao Y, Pu JX, Huang SX, Wu YL, Yang LB, et al. (2009) Ent-kaurane diterpenoids from *Isodon scoparius*. J Nat Prod 72: 125–129.

84. Jiang B, Mei SX, Zhao AH, Sun HD, Lu Y, et al. (2002) Diterpenoids from *Isodon sculponeatus*. Chin J Chem 20: 887–890.

85. Li LM, Li GY, Ding LS, Lei C, Yang LB, et al. (2007) Sculponins A-C, three new 6,7-seco-ent-kauranoids from *Isodon sculponeatus*. Tetrahedron Lett 48: 9100–9103.

86. Li LM, Li GY, Pu JX, Xiao WL, Ding LS, et al. (2009) Ent-kaurane and cembrane diterpenoids from *Isodon sculponeatus* and their cytotoxicity. J Nat Prod 72: 1851–1856.

87. Lia X, Pu JX, Weng ZY, Zhao Y, Zhao Y, et al. (2010) 6,7-seco-ent-kaurane diterpenoids from *Isodon sculponeatus* with cytotoxic activity. Chem Biodivers 7: 2888–2896.

88. Takeda Y, Takeda K, Fujita T, Sun HD, Minami Y (1990) Studies on the diterpenoid constituents of *Rabdosia ternifolia* - Structural elucidation of new diterpenoids, rabdoternin-A, rabdoternin-B and rabdoternin-C. Chem Pharm Bull 38: 439–442.

89. Takeda Y, Takeda KI, Fujita T, Sun HD, Minami Y (1994) Rabdoternins D-G, ent-7-beta,20-epoxykaurenes from *Rabdosia ternifolia*. Phytochemistry 35: 1513–1516.

90. Wang ZM, Feng H, Liang XT (1996) Structures of isodoternifolin A and B. Chin Chem Lett 7: 816–817.

91. Wu SH, Zhang HJ, Chen YP, Lin ZW, Sun HD (1993) Diterpenoids from *Isodon wikstroemioides*. Phytochemistry 34: 1099–1102.

92. Hou AJ, Li ML, Jiang B, Lin ZW, Ji SY, et al. (2000) New 7,20 : 14,20-diepoxy ent-kauranoids from *Isodon xerophilus*. J Nat Prod 63: 599–601.

93. Hou AJ, Zhao QS, Li ML, Jiang B, Lin ZW, et al. (2001) Cytotoxic 7,20-epoxy ent-kauranoids from *Isodon xerophilus*. Phytochemistry 58: 179–183.

94. Li SH, Niu XM, Peng LY, Zhang HJ, Yao P, et al. (2002) Ent-kaurane diterpenoids from the leaves of *Isodon xerophilus*. Planta Med 68: 946–948.

95. Li LM, Weng ZY, Huang SX, Pu JX, Li SH, et al. (2007) Cytotoxic ent-kauranoids from the medicinal plant *Isodon xerophilus*. J Nat Prod 70: 1295–1301.

96. Weng ZY, Huang SX, Li ML, Zeng YQ, Han QB, et al. (2007) Isolation of two bioactive ent-kauranoids from the leaves of *Isodon xerophilus*. J Agric Food Chem 55: 6039–6043.
