# Supplementary figures and images for: Selection for High Oridonin Yield in the Chinese Medicinal Plant Isodon (Lamiaceae) Using a Combined Phylogenetics and Population Genetics Approach
Source: PLoS One. 2012 Nov 27;7(11):e50753. doi: 10.1371/journal.pone.0050753 (PMC3507737; doi:10.1371/journal.pone.0050753)

**Table S4. Representative chromatograms of *Isodon rubescens* and *Isodon japonicus***


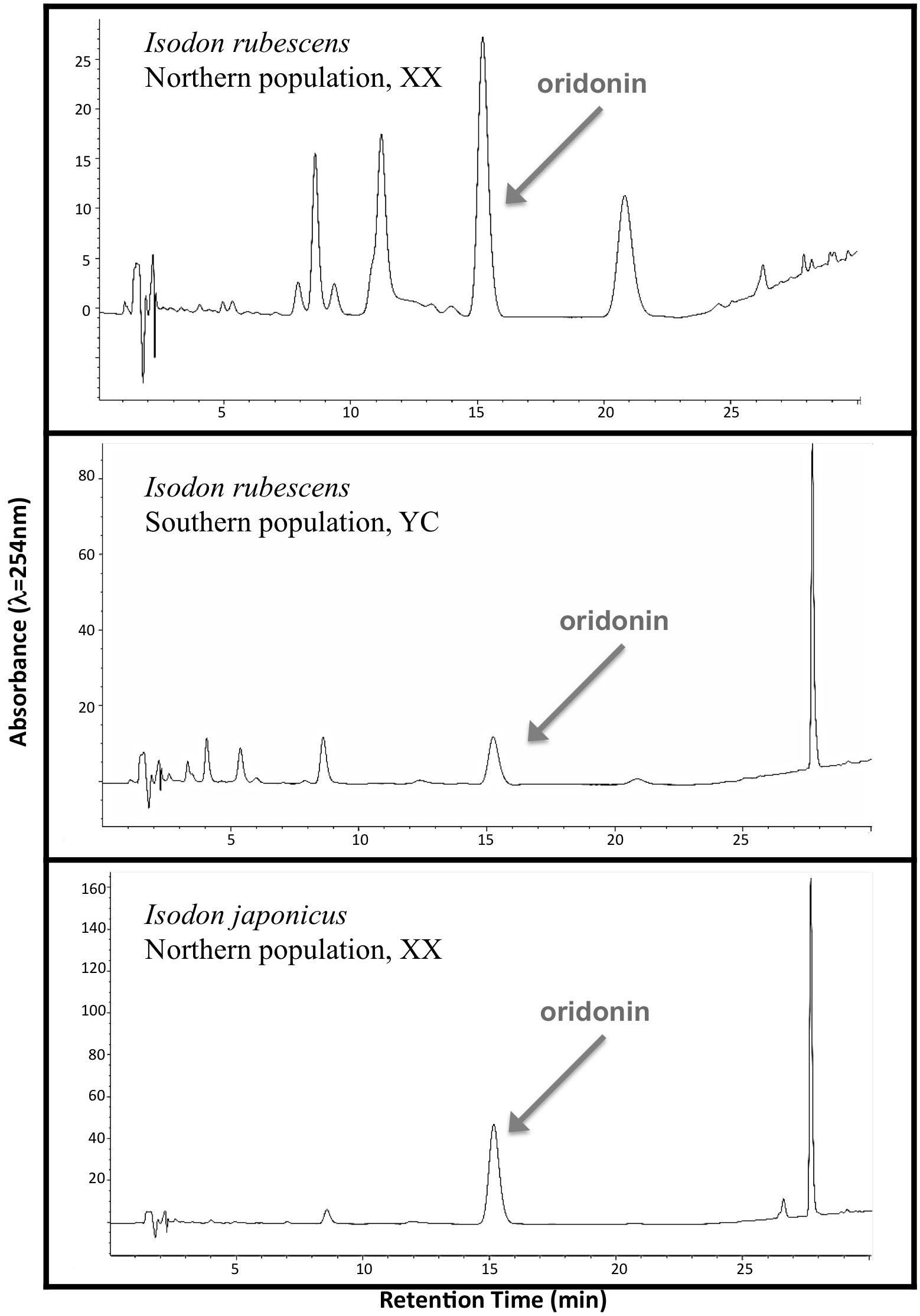

Supplement: Table S4 — Representative chromatograms of Isodon rubescens and Isodon japonicus . (DOC) [file pone.0050753.s004.doc]
